# Cadmium and Kidneys: Low-Level Exposure and Effects in Women

**Published:** 2005-11

**Authors:** Julia R. Barrett

Widespread exposure to the heavy metal cadmium occurs through both natural and industry-related sources. The general population is likely to encounter low-level chronic exposure through smoking and from dietary sources, particularly shellfish, grains, and vegetables. In 1999 an ongoing population-based Swedish study, Women’s Health in the Lund Area, was expanded to include low-level cadmium exposure. Analysis of the data collected now reveals a small but significant kidney response to low-level cadmium exposure **[*EHP* 113:1627–1631]**. This suggests that low-level cadmium exposure may pose a significant public health risk.

Owing to extremely slow excretion, cadmium accumulates in the body, especially in the kidneys. Kidney damage is the primary consequence, but most toxicity data are from exposures in occupational settings or severely polluted areas. The effects of low-level exposure are less certain.

A primary function of the kidney is to filter excess water and metabolic by-products from the blood for urinary excretion. This filtration occurs in more than 1 million nephrons, each of which contains a blood capillary (the glomerulus) intertwined with a urine-collecting tubule. In the current study, researchers assessed glomerular and tubular fitness by measuring kidney function markers in blood and urine, respectively. Blood testing also revealed ongoing cadmium exposure, and urinalysis indicated cadmium body burden.

The team analyzed data, including blood and urine samples, collected from 820 women aged 54–63 years. Blood levels of creatinine and cystatin C were measured in 742 participants to calculate glomerular function. Urinary concentrations of calcium, human complex-forming protein, and *N*-acetyl-β-d-glucosaminidase—all markers of tubule function—were available for 813 women. The researchers additionally collected data on medications taken, smoking history, lead exposure, and incidence of diabetes and hypertension to control for potential confounding factors.

Cadmium concentrations were similar or slightly higher compared with previous data from Sweden and much lower than concentrations reported for populations in highly polluted areas in Europe and Japan. Current or former smokers had cadmium concentrations that were 90% higher in blood and 40% higher in urine than concentrations measured in participants who never smoked. Consequently, multivariate analyses were conducted on data from all participants as one group and from those who had never smoked as another group.

Cadmium concentrations were positively associated with the tubular function markers, indicating some damage to the tubules. Increased cadmium was also associated with decreased creatinine clearance, reflecting a reduced glomerular filtration rate. The lowest-observed-effect level for increased tubular markers was a mean urinary cadmium concentration of 0.6 microgram per liter, which is lower than previously reported. A reduction in glomerular filtration rate was associated with a minimum mean urinary cadmium concentration of 0.86 microgram per liter.

The researchers speculate that effect levels might be even lower for people with diabetes, a disease carrying high risk of kidney damage similar to that caused by cadmium exposure. Although the effects of low-level cadmium exposure are clinically minor, they should be viewed as early indicators of potential severe health effects, according to the researchers. Given the size of the exposed population, there may be a significant public health risk, and efforts beyond smoking cessation programs are needed to reduce exposure.

## Figures and Tables

**Figure:**
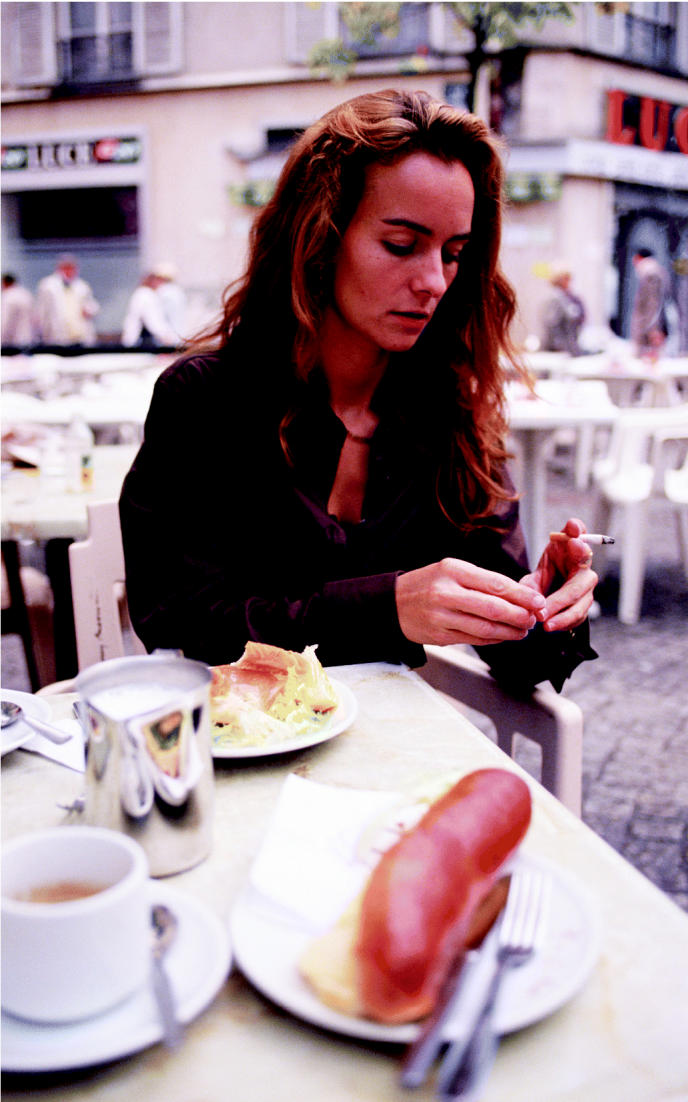
Cadmium connection. A new study shows that kidneys respond to even low-level chronic cadmium exposure such as that obtained from smoking and eating grains.

